# Knowledge, attitudes and practices of Iranian people about food safety and hygiene during covid-19 pandemic

**DOI:** 10.1186/s12889-022-13559-1

**Published:** 2022-06-08

**Authors:** Ali Salehi, Fatemeh Salmani, Ensiyeh Norozi, Parisa Sadighara, Tayebeh Zeinali

**Affiliations:** 1 Department of Environmental Health, Food Safety Division, School of Public Health, Tehran University of Medical Sciences, Tehran, Iran; 2 Department of Epidemiology and Biostatistics, School of Health, Social Determinants of Health Research Center, Birjand University of Medical Sciences, Birjand, Iran; 3 Department of Public Health, School of Health, Social Determinants of Health Research Center, Birjand University of Medical Sciences, Birjand, Iran

**Keywords:** Knowledge, Attitude, Practice, Validity, Factor analysis, Food safety

## Abstract

**Aim:**

The objective of this study was to develop a cultural adopted questionnaire for evaluation of knowledge (K), attitude (A) and practice (P) of Iranian population toward food safety during Covid-19.

**Methods:**

The study is based on an online questionnaire that filled by 712 Iranians over 16 years old. Exploratory factor analysis (EFA), confirmatory factor analysis (CFA) and reliability assessment were performed. The construct validity of A and P determined by EFA and confirmed by CFA. Difficulty index was used for K.

**Results:**

The reliability score of questionnaire was satisfactory. The three items of K-A-P questionnaire were significantly associated with the total score of questionnaire. The KAP questionnaire regarding food safety in covid-19 consisted of 27 items multidimensional scale with strong psychometric features. The respondent showed a satisfactory level of KAP during covid-19 pandemics.

**Conclusion:**

The KAP questionnaire regarding food safety in covid-19 is a valid and reliable tool for measurement of knowledge, attitude and practice of people regarding food safety in covid-19.

## Introduction

Coronaviruses are a wide group of viruses that often cause mild to severe respiratory tract infections like the common cold [1]. Over the last two decades, three novel coronaviruses have evolved from animal reservoirs, causing significant and widespread illness and death [2]. There are hundreds of coronaviruses, the majority of which are found in animals such as pigs, camels, bats, and cats. 229E NL63 OC43 HKU1 SARS-CoV MERS-CoV and SARS-CoV-2 are the seven varieties of known coronaviruses that cause illness in humans [3]. In December 2019, SARS-CoV-2 appeared from Wuhan, China, causing COVID-19 illness. On March 11, 2020, the World Health Organization (WHO) labeled this disease a worldwide pandemic. Approximately two-thirds of the primary cases of patients were found to be linked to the Huanan seafood market in Wuhan, where in addition to aquatic animals, other animals such as civets and raccoon dogs were also sold live [4]. Although, reputable international organizations, including the WHO, food and drug Administration (FDA) and the center for disease control (CDC), have not reported any evidence of covid-19 transmission by food, many people are concerned about covid-19 transmission through water and food due to reports of primary cases of the disease from the seafood market [5].

According to the literature, SARS-CoV-2 is a respiratory virus that spreads directly through infected people's respiratory droplets when they sneeze or cough [6]. The modes of transmission of this virus differ from those of food-borne viruses such as noroviruses and hepatitis viruses. However, it is possible to transmit it indirectly through an infected person working in food establishments. In other words, if an infected person touches the food, sneezes or coughs on it, and then another person comes into contact with the same food and touches his/her nose, eyes, or consumes it, there is a potential of transmission [7]. Furthermore, because studies have shown that the SARS-CoV-2 can survive in cold and refrigerated environments, there is a chance that the virus will be present in refrigerated and cold foods [8].

Despite the start of worldwide vaccination and the proportion of good coverage in communities, the morbidity and mortality rate remain high. As a result, in order to control and eliminate this disease, people must continue to follow health and safety protocols (physical and social distance, masks, etc.) in addition to vaccination [9].

The way people behave in society has a direct impact on the control and prevention of Covid 19 disease, and the process of transmission of this disease is affected by public health behaviors. The existence of preventive health behaviors in times of epidemic in society requires a high level of health knowledge and attitude. The knowledge and attitudes of people about health concerns and issues can lead to the prediction of a health behavior [10]. According to studies, increasing people's knowledge about health issues has a highly favorable effect on improving health status and leading to healthier attitudes and practices. For instance, Jahed Khaniki et al. (2012) conducted a research at Tehran University of Medical Sciences to assess students' knowledge and attitudes on health and food safety. They found that students who have completed food-related courses had a very high level of knowledge and attitude towards other students, as well as significantly better health practices [11].

Given the importance of people's knowledge and attitude regarding food safety during Covid pandemic and absence of related studies among Iranian people, the aim of this study was to determine the level of knowledge, attitude and practice of Iranian peoples (More than 16 years old) about food safety and hygiene during the Covid-19 disease pandemic using an electronic questionnaire.

## Materials and methods

### Study design

A descriptive-analytical study was conducted using an online questionnaire to investigate Iranian peoples' knowledge (K), attitude (A), and practice (P) about food safety and hygiene during the Covid-19 pandemic. The questionnaire was distributed to many various groups such as family, academic, industrial, general, specialized, etc. using the E-mail and most popular social media platforms in Iran, including Instagram, Whats-app, Telegram, and Facebook. The online survey was conducted from 25 August 2021 to 24 September 2021. The IP address of the questionnaire was designed in such a way that participants could only responds once and anonymously. After the end of the response time, the raw data were automatically collected in an Excel file.

### Design of questionnaire

The questionnaire was divided into four sections and was written in Persian. The first section included demographic information such as age, gender, marital status, place of residence, level of education, occupation, income status, history of chronic illness, and how to become acquainted with health and food safety issues. Part two focused on participants' knowledge about food safety and hygiene during the Corona outbreak, which was evaluated using true and false responses. In the third part participants' attitudes on food safety and hygiene evaluated. The answers to the questions in this section were considered as five point Likert scale (strongly agree, agree, neither agree nor disagree, disagree and strongly disagree). Part four was related to hygienic practice of participants during the Corona outbreak and was measured by five point Likert scale (never, rarely, sometimes, often, and always).

### Item generation

Two approaches were used for item generation, including review of previous published literature and opinions of focus groups to obtain understanding of the potential knowledge, attitudes and behavior regarding food safety during Covid19 pandemic. Totally 38 initial items were generated which include 8, 18 and 13 items for knowledge, attitude and practice, respectively. Knowledge and attitude items were included individual’s knowledge and beliefs, respectively, about food safety aspect of corona virus. Items of behavior measured the individual’s hygienic practice during preparation and cooking of food in covid19 pandemic.

### Face and content validity

The face validity of the initial items was assessed by taking the opinions of target population about clarity and easiness of each item. The unclear and difficult items were rephrased and reworded. To assess the validity, the designed questionnaire was given to a panel of ten experts including health educators, epidemiologists, biostatistics, nutritionist, and food safety and hygienist, and content validity was quantified using the Content Validity Ratio (CVR) and Content Validity Index (CVI) according to Lawsche table [12]. To calculate the CVR, experts appraised the necessity of each item on a three-point Likert scale (necessary, helpful but not necessary and not necessary). In addition, to calculate the CVI, the experts scored each item on a four-point scale based on its simplicity, relevance, and clarity. CVR and CVI were acceptable if they were greater than 0.62 and 0.78 [12].

### Sampling method & Sample size calculation

Inclusion criteria was including, the minimum of 16 years old age, enable to read and write, having an internet connection and smart phone. There was no specific exclusion criterion. Sampling was carried out by using snowball sampling method. Every person who filled the questionnaire was asked to send it to his/her friends, relatives or contacts and etc. until the sample size was completed. Since the data was gathered through virtual social media, the questionnaire link was distributed in social, family, academic, industrial, general and specialized groups following correspondence and coordination with the Student Research Committee, food safety and hygiene association, and the Women's Affairs of the Governorate.

Based on the Shi et al. study, the sample size was calculated using the following formula: α, β and d were considered as 0.95, 0.8 and 0.17 respectively and the sample size was obtained 661. In the current study, 712 questionnaires were collected virtually.

$$n = \,\frac{{(z_{{1 -_{2}^{a} }} + \,z_{1 - \beta } )^{2} \sigma^{2} }}{{d^{2} }}$$

The study was approved by the Birjand University of Medical Sciences ethics committee (IR.BUMS.REC.1400.184). Accordingly, the aim of the study was explained to the participants, and confidence was given about anonymous and voluntary nature of the study in the questionnaire.

### Construct validity

#### Knowledge; difficulty index (Sample: *n* = 712)

The difficulty index of knowledge items of the questionnaire was assessed according to following equation [13]:

$$Item\,Difficulty\,Index\, = \,\frac{Number\,of\,currect\,responses\,to\,each\,knowledge\,item}{{Total\,number\,of\,responses\,(both\,correct\,and\,incorrect)}}$$

#### Attitude and practice; exploratory factor analysis (calibration sample: *n* = 356):

To assess the factor structure of the questionnaire, exploratory factor analysis (EFA) was performed using the SPSS software V.23 on a random splithalf sample of the data. The Kaiser–Meyer–Olkin (KMO) test was also used to evaluate the adequacy of the sample size. KMO value ≥ 0.70 was considered as criterion for sampling adequacy [12]. The factor structure of questionnaire was explored by Principal Axis Factoring with Varimax rotation by Maximum Likelihood method. Kaiser’s criteria (eigen value > 1 rule), and the Scree plot [14], were used as main criteria for verifying the factor structure.

#### Attitude and practice; confirmatory factor analysis (calibration sample: *n* =356)

Multiple fit indices and cut-offs were used to assess the goodness of fit of the data, including the Tucker–Lewis Index [15] with a cut-off value of TLI ≥ 0.90, Comparative Fit Index (CFI) with a cut-off value of CFI ≥ 0.90, Root Mean Squared Error of Approximation (RMSEA) with a cut-off value of RMSEA ≤ 0.08, the normed χ2 with a cut-off value of normedχ2/df < 5 and Parsimonious Normed Fit Index (PNFI) with a cut-off value of PNFI ≥ 0.5 were used [16, 17]. All these tests were performed using LISREL software (version 8.8). Goodness-of-Fit-Index (GFI) by cut of value ≥ 0.9 and Parsimonious Comparative Fit Index (PCFI) by cut of value ≥ 0.6 were also performed [18].

### Reliability assessment

In order to investigate the reliability of the questionnaire, the Kuder-Richardson-20 (KR-20) for knowledge and Cronbach's Alpha coefficient for attitude and practice were used. They were performed on 10 people that were not included in the final analysis. Value more than 0.70 was considered to assess the scale's reliability [19].

### Statistical analysis

Data was analyzed using SPSS software V.23. The mean and standard deviation indices were used to description of data. Correlation of different items of the questionnaire was taken by Pearson analysis. Significance level was considered < 0.05.

## Results

### Face and content validity

Among 38 initial items, 6 of them (three of A and three of P) had some difficulty in comprehension and were deleted. CVR and CVI of remaining 32 items were according to Marsh, 1988 and showed high content validity.

### Construct validity

**Fig. 1:**
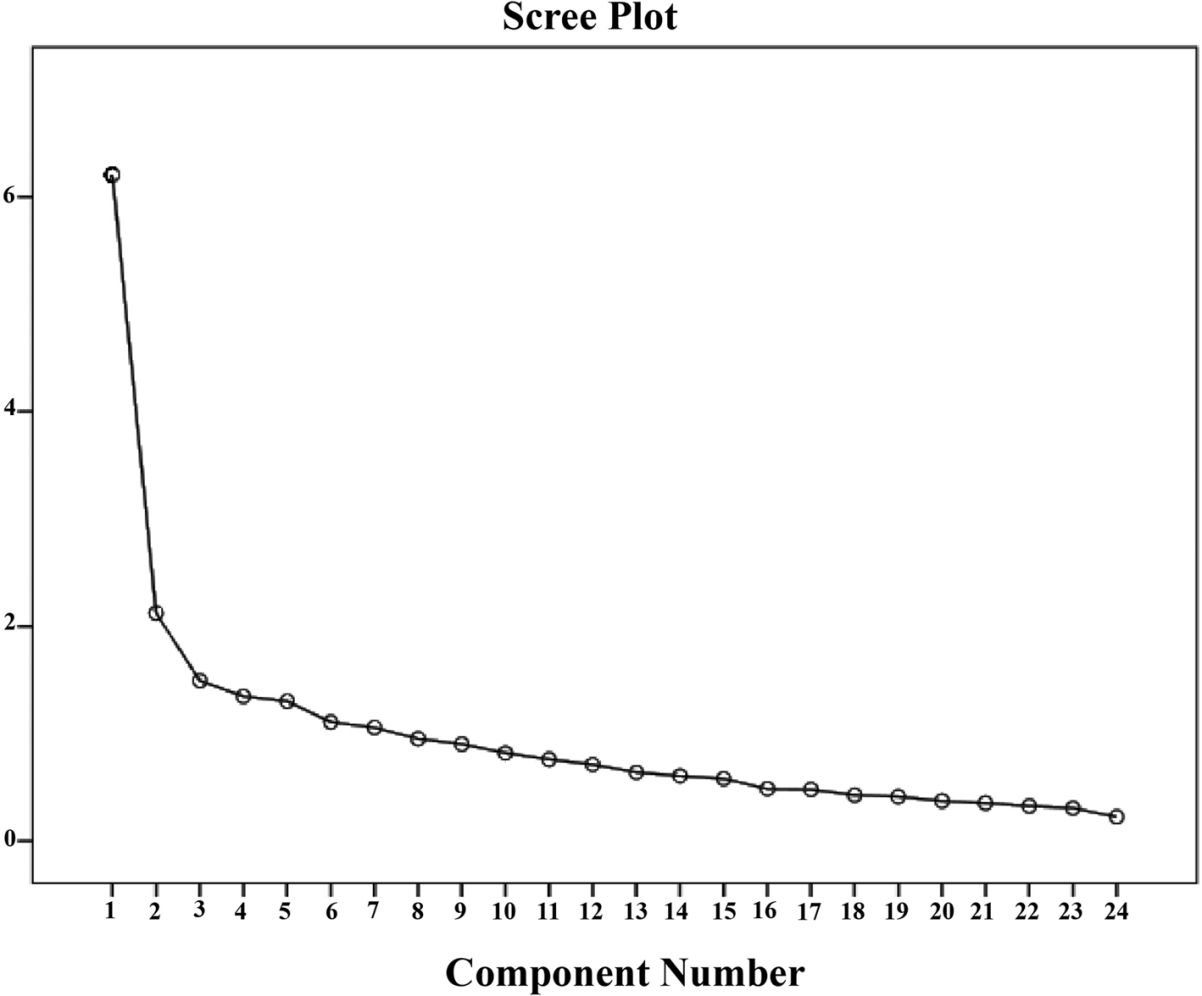
Scree plot of loading factors of 24 items of attitude and practice toward food safety in Covid19 in Iranian population

Table 1 shows the difficulty index of the knowledge questions. EFA and CFA for attitude and practice questions were performed. Deletion of unrelated items was carried out in EFA. The KMO for sampling adequacy was 0.85. A significant correlation was existed between the questions by Bartlett's test (χ2: 2683.77, *p* < 0.001). Scree plot shows the two factors (Fig. 1). Varimax rotation method in principal component factor analysis resulted to 20 items questionnaire that categorized in two factors of attitude and practice (Table 2). In fact, four items were not suitably grouped in designed factors. First factor, attitude, included ten items, and second factor, practice contained 10 items. The total variance explained by attitude, and practice were 16.77% and 17.91%, respectively and ensemble was 34.68%. High inter-correlation was seen between factors and total score of questionnaire. The CFA through chi-square test was significant (Chi-Square = 632.44, *P* < 0.001). A good fit to the data in the model was acquired through root mean square residual (normedχ2/df = 2.14, GFI = 0.92, TLI = 0.90, CFI = 0.92, PNFI = 0.71, PCFI = 0.76, and RMSEA = 0.057).

**Table 1:** Difficulty index of the knowledge items

	questions	difficulty index
1	The corona virus survives on skin, nasal mucosa and saliva	87.6%
2	Handwashing before cooking can be prevented the transmission of COVID-19	94.8%
3	Raw and cooked foods should be kept separate in the refrigerator	91.4%
4	The Corona virus **does not** survive in the freezer	20.4%
5	The corona virus inactivated at cooking temperature (above 60° C)	77.2%
6	The cutting board of raw and cooked food should be separated	87.1%
7	Corona virus **transmits** through eating food	50.7%

**Table 2:** Exploratory factor analysis of 20 items of attitude and practice toward food safety in Covid19 in Iranian population

Questions	Factor’s Loading	
	Attitude	Behavior
I'm in good health and I'm unlikely to get Covid-19	.098	
I believe that Covid-19 is a very serious disease	.403	
If I do not follow the hygiene tips during shopping I will get covid-19	.762	
If I do not follow the hygiene tips during cooking I will get covid-19	.761	
By adherence to health protocols during shopping and cooking of foods, I can prevent transmission of Covid-19 to others	.673	
from my perspective, COVID-19 is not related to foods	.582	
from my perspective, disinfecting food packaging is futile	.586	
It gives me a sense of tranquility and security by following health protocols related to food safety	.580	
There is a possibility of catching covid-19 at any stage from shopping to preparation and cooking of food	.738	
Even the thought of having Covid-19 scares me	.288	
Do you clean your hands with soap before preparing and consumption of food?		.470
If you do food shopping, do you follow health protocols (gloves, masks, social distance) during food shopping?		.639
Do you use foods such as egg, chicken and fish in cooked form?		.622
Do you disinfect food packaging before opening?		.567
Do you cleaning food contact equipment and surfaces on a daily basis?		.569
Do you store raw and cooked foods separately?		.599
Is the cutting board of raw and cooked food separate?		.607
Do you reheat food leftovers before eating it?		.428
Do you use separate utensils for each person when serving food during Corona?		.471
If you do food shopping, do you avoid going to crowded shopping malls to groceries Shopping?		.632

### Reliability assessment

The KR-20 for knowledge and Cronbach's Alpha coefficient for attitude and practice were 0.636, 0.65 and 0.79, respectively. Accordingly, the reliability of practice was confirmed, however knowledge and attitude had lower value of KR-20 and Cronbach’s alpha. In order to increase them, one item of knowledge and one item of attitude were deleted.

### Demographic characteristics of study population

The mean age of the participants was 38.88 ± 9.56 (mean ± standard deviation (SD)). The minimum and maximum of age of participants were 16 and 74 years, respectively. Most of the participants were female (63.3%) and married (74.9%). Education of participants was mostly Bachelor of Science/Art (40.7%) and Master of Science/Art (32%). 28.7% of the participants had a monthly income of 30 to 70 million Rials (IRR) and 46.5% said that had a medium income. Most of the participants had corporation in shopping (89.7%) and cooking (81.9%) of food. 34.1% and 26.1% of population gathered information regarding food safety through reliable references and academic degrees. Most of the participants (86.9%) had not any chronic illness. Table 3 presents the demographic characteristics of study population.

**Table 3:** Demographic characteristics of study population

Variables		Frequency (Percent)
Sex	Male	261 (36.7%)
	Female	451 (63.3%)
Status	Single	179 (25.1%)
	Married	533 (74.9%)
Education	Lower than High school	29 (4.1%)
	High school	55 (7.7%)
	Technician/ Bachelor of Science/Art	290 (40.7%)
	Master of Science/Art	228 (32%)
	Ph.D	110 (15.4%)
Income (IRR)	< 10 m	92 (13.5%)
	10–30 m	86 (12.6%)
	30–70 m	196 (28.7%)
	70–100 m	177 (25.9%)
	> 100 m	133 (19.4%)
Income evaluation	Low	316 (44.4%)
	Middle	331 (46.5%)
	Good	65 (9.1%)
Corporation in shopping of food	No	73 (10.3%)
	Yes	639 (89.7%)
Corporation in cooking of food	No	129 (18.1%)
	Yes	583 (81.9%)
Gathering information regarding food safety	Nothing	32 (4.5%)
	Related to academic degree	186 (26.1%)
	Related to job	107 (15%)
	News	144 (20.2%)
	Reliable References	243 (34.1%)
Chronic illness	No	619 (86.9%)
	Yes	93 (13.1%)

### Knowledge, attitude and practice of Iranian population regarding food safety in Covid19

Measurement of reliability of the questionnaire was performed for a total of 712 study participants. Table 4 shows the reliability score, and descriptive statistics relation of three items of the questionnaire. Relation of the three parts was assessed (Table 4). The three domains were significantly related with the total score of questionnaire (*p* < 0.001) (Table 4). A significant association was seen between attitudes and behavior and also attitudes with knowledge (*p* < 0.001).

**Table 4:** Mean, SD and reliability scores of the questionnaire (Calibration sample: *n* = 712)

Domain	Minimum	Maximum	Mean	SD	Skewness	Kurtosis	Reliability score	Relation with Practice
Knowledge	0.86	2	1.54	0.24	-0.25	-0.13	0.82	0.15 (p < 0.001)
Attitudes	1.22	5	3.87	0.59	-0.73	1.07	0.74	0.48 (p < 0.001)
Practice	1.00	5	4.38	0.56	-1.55	4.28	0.80	-

## Discussion

In this study, we developed a valid and reliable questionnaire to assess the knowledge (K), attitude (A) and practice (P) of Iranian peoples regarding food safety during covid-19 pandemics. Because of multidimensional nature of human activity during pandemics developing comprehensive questionnaires, which contain the important aspect of individual’s relationship and activity, is an urgent need [20]. The developed questionnaire by our team included the essential items to evaluate the personal, interpersonal and the functional activity of individual in society during pandemics.

We evaluate the knowledge of individuals about covid-19 pandemic based on difficulty index. The difficulty index also was satisfactory. Based on our results from KMO test in EFA analysis the sample size of our study was sufficient. During the EFA analysis 20 question were included in the two main groups of our questionnaire. Different item indicators were used to evaluate the dimension of the designed questionnaire. The GFI was in the acceptable range [21]. Different research articles showed that GFI could be influenced by different factors such as degree of freedom and sample size [22, 23]. Our results showed that χ2/df of our model were also within the range of recommended value. The Parsimony-Adjusted Measures Index (PNFI) was in the acceptable range of model fit. One of most preferred measures of model fit is RMSEA. The value exhibits that the developed model was acceptable. According to the results of the CFA analysis, the items were fit for the expected scale. The mentioned CFA indices also confirmed our developed model. Based on the results of descriptive statistics the KR-20 of knowledge and Cronbachs’ alpha of attitude and practice were sufficient.

A Specific covid-19 K-A-P questionnaire was designed by park in 2021. This questionnaire was developed based on international and domestic information. Ten experts evaluated the items and determined the content validity. The Kaiser–Meyer–Olkin value of 0.735 of this questionnaire indicated a highly acceptable score. The Cronbach's α of 0.75 showed an acceptable level of reliability of their tool [24]. Our designed tool is in agreement with her work. The Cronbach's Alpha for attitude and practice of our tool were 0.74 and 0.80 respectively. KR-20 results also indicated a 0.82 score for knowledge aspect of our questionnaire. These results indicate that K-A-P measurement instrument is a valid tool to assess the knowledge, attitudes and practices about covid-19.

Zhong and his colleagues designed another K-A-P measurement tools. Their instrument consisted of two parts including demographic information and KAP. Their questionnaire had 12 question including four clinical presentations (K1-K4), three transmission routes (K5-K7), and five prevention and control (K8-K12) of COVID-19. The Cronbach's alpha coefficient of the knowledge questionnaire was 0.71 in their sample, indicating acceptable internal consistency [25]. Their tool internal consistency is less than our knowledge questionnaire, which is 0.82 and a higher reliability of our instrument.

Results of current study showed that Iranian people had a high level of knowledge, attitude and practice regarding food safety in Covid-19. In this study, 88.2% of the participants had a college or university degree. These findings demonstrate the importance of level of education in enhancing knowledge and attitudes, which leads to successful hygienic practice performance. According to statistical analysis, more than 95% of participants gathered information regarding food safety through various media. These findings indicate that people has felt the need to learn more information during the corona outbreak, and getting this information naturally has led to increased knowledge, attitude, and, as a result, better hygienic practice performance. The most important reason is related to the measures taken by Iran ministry of health and medical education, including sensitization, virtual training, advertising and installation of educational banners in public view, encouraging people to use packaged food and etc.

Several researches have been conducted to evaluate the knowledge, attitudes, and practices of working people in food industry worldwide during covid-19. Jubayer and his colleuges designed a questionnaire to assess the knowledge, attitude, and practices of food industry workers in Bangladesh and their results were promising. The correct response rate of their model were for knowledge 89.7%, attitude 93%, practices 88.2% and they concluded that the respondent showed a satisfactory level of KAP during covid-19 pandemics [26]. Another article performed in Egypt by Abdelrassoul and colleagues. They assessed the knowledge, attitude, and performance of home service providers (motor couriers) of fast food restaurants in Egypt. Six hundred paper questionnaires were distributed among food delivery providers. Their findings showed that people have good knowledge and awareness. However, the level of performance in these persons was low and they were not able to use their knowledge to serve the customers during the covid-19 pandemic. They showed that in order to increase and improve the functional behavior of service providers, continuous training, monitoring and supervision should be done and the existence of a control team for coronary heart disease in restaurants is necessary [27].

Li et al. (2020) in their online questionnaire study assessed and evaluated the knowledge, attitude and practice of more than 123,000 workers in various factories about the new coronavirus in China. In this cross-sectional study, questionnaires were collected online. There were 20 questions about knowledge, six questions about attitude and six questions about performance of workers. The mean scores of knowledge, attitude and practice were 16.3, 4.5 and 5.8, respectively. In this study, about 68.8% of workers positively evaluated gargling with salt water to protect against Covid-19 disease. The study also found that, on average, older and less educated people had low knowledge and practice about Covid-19 disease [28]. Shi and Zhang performed another research in china about the subject. They examined the effects and consequences of Covid disease on the knowledge and practice of food consumers about food safety in their study, data were collected from 1373 participants in the form of an electronic questionnaire. Their findings show that during the Covid-19 epidemic, people paid more attention to health protocols [29].

Luo performed a survey to investigate the knowledge, attitudes and practices (K-A-P) of Chinese people about food safety during covid-19 pandemics. Their designed questionnaire included socio-demographic information, the attention towards COVID-19, K-A-P about food safety and nutrition. Total number of 2272 participant was included in this study. The results of this article indicated that K-A-P of Chinese individuals should be more improved during covid-19 pandemic due to health emergency. They also suggested that other measures such as improving educational materials about covid-19 should be implemented in order to avoid biased or misleading information [30]. With the emergence of the Covid-19 epidemic, the Iranian Ministry of Health and medical education required all citizens to follow health guidelines in order to control and prevent the irreversible repercussions of Covid-19 disease. As a result of these initiatives, people became more aware of the protocols. Undoubtedly, the Covid-19 pandemic influenced people's knowledge and behavior towards food safety. Our findings in this study showed that the Iranian people in the context of the Covid-19 epidemic had a high level of knowledge, attitude and behavior about food hygiene and safety. In other words, it is possible to claim that knowledge, attitudes and practices of people have improved during the covid-19 pandemic due to health emergency conditions.

## Conclusion

Our study introduced a specific KAP regarding food safety in covid-19 questionnaire consisted of 27 item multidimensional scale with strong psychometric features. This designed tool is a valid and reliable questionnaire, which allows the researcher to evaluate the knowledge, attitudes and practices of the participants regarding food safety during covid-19 pandemics. This instrument has some advantage compare to other developed questionnaire mentioned above due to culturally adoption and could be potent asset to provide better understanding of knowledge, attitudes and practices during covid-19 pandemics. Moreover, our findings showed a satisfactory level of KAP during covid-19 pandemics. As the study was an online survey, people who have able to fill the online questionnaire participated in this study. Due to the changing situation of Covid pandemic we need to gather data in a limited period.

## Data Availability

The datasets used and/or analysed during the current study are available from the corresponding author on reasonable request.

## Abbreviations

EFA: Exploratory Factor Analysis
CFA: Confirmatory Factor Analysis
K: Knowledge
A: Attitudes
P: Practice
CVR: Content Validity Ratio
CVI: Content Validity Index
KMO: Kaiser–Meyer–Olkin
TLI: Tucker–Lewis Index
CFI: Comparative Fit Index
RMSEA: Root Mean Squared Error of Approximation
PNFI: Parsimonious Normed Fit Index
GFI: Goodness-of-Fit-Index
PCFI: Parsimonious Comparative Fit Index
